# Associations between the size and duration of asymptomatic subchorionic hematoma and pregnancy outcomes in women with singleton pregnancies

**DOI:** 10.1186/s12884-023-05831-y

**Published:** 2023-08-02

**Authors:** Shuangjia Pan, Yehui Lan, Yujia Zhou, Baoyi Chen, Feifei Zhou, Dongru Dai, Ying Hua

**Affiliations:** 1 Department of Obstetrics and Gynecology, the Second Affiliated Hospital of Wenzhou Medical University, Wenzhou, 325027 China; 2 Department of Obstetrics and Gynecology, the Wenzhou Third Clinical Institute Affiliated to Wenzhou Medical University, Wenzhou, 325000 China

**Keywords:** Subchorionic hematoma, Pregnancy outcomes, Singleton pregnancies, Size of hematoma, Duration of hematoma

## Abstract

**Background:**

The purpose of this study was to investigate the relationship between the size and duration of asymptomatic subchorionic hematoma and pregnancy outcomes in women with singleton pregnancies.

**Methods:**

This was a retrospective study that enrolled 701 singleton pregnant women who were diagnosed with asymptomatic subchorionic hematoma by ultrasound at 5–10 gestational weeks. The control group recruited 640 normal pregnant women without subchorionic hematoma who were matched with subchorionic hematoma group on baseline characteristics. The pregnancy outcomes were compared between the two groups, and the associations of the size and duration of subchorionic hematoma with pregnancy outcomes were analyzed by logistic regression model.

**Results:**

Compared with the normal pregnancy group, the incidence of, gestational diabetes mellitus, gestational thrombocytopenia, placenta adhesion, fetal growth restriction, macrosomia in subchorionic hematoma group were higher (all *P* < 0.05). After adjusting for confounding factors, the hematoma size was positively associated with the occurrence of gestational hypothyroidism (adjusted OR[95%CI]: 1.029[1.004–1.054]), intrahepatic cholestasis of pregnancy (adjusted OR[95%CI]: 1.095[1.047–1.146]), term premature rupture of membranes (adjusted OR[95%CI]: 1.044[1.005–1.085]), hypertensive disorders of pregnancy (adjusted OR[95%CI]: 1.030[1.0004-1.060]), gestational thrombocytopenia (adjusted OR[95%CI]: 1.078 [1.045–1.113]), placenta adhesion (adjusted OR[95%CI]: 1.054 [1.027–1.082]), and the duration of hematoma was positively associated with the incidence of term premature rupture of membranes (adjusted OR[95%CI]: 1.070[1.027–1.115]), gestational diabetes mellitus (adjusted OR[95%CI]: 1.938 [1.886–1.993]) and fetal growth restriction (adjusted OR[95%CI]: 1.194 [1.124–1.268]).

**Conclusions:**

The presence, size and duration of a first-trimester asymptomatic subchorionic hematoma may be associated with adverse pregnancy outcomes at later gestations such as term premature rupture of membranes and fetal growth restriction.

## Introduction

Subchorionic hematoma (SCH), being commonly observed on ultrasound examinations during the first trimester, usually appears as a hypoechoic or anechoic crescent-shaped area between the chorionic membrane and the myometrium in both asymptomatic patients and those presenting with vaginal bleeding [1, 2]. The reported incidence of subchorionic hematoma varies widely from 1.3–39.5% [3, 4]. Some pregnancies with subchorionic hematoma present with vaginal bleeding [1]. However, with the popularization of ultrasound in early pregnancy, the incidence of asymptomatic subchorionic hematoma has increased significantly.

Poor placentation can impair angiogenesis, leading to the formation of weak vessels that prone to tearing, which is believed to be the underlying cause of subchorionic hematoma [5, 6]. Placental dysfunction is also one of the mechanisms involved in threatened abortion and a series of complications in late pregnancy such as preterm birth, premature rupture of membranes (PROM), intrauterine growth restriction (IUGR), and preeclampsia [7, 8]. Therefore, subchorionic hematoma in the first trimester may be associated with perinatal complications, and it may identify a population of patients at increased risk of adverse pregnancy outcomes.

However, the association of subchorionic hematoma with adverse pregnancy outcomes remains inconclusive based on the limited data [6, 9, 10], with some studies demonstrating increased rates of complications like pregnancy loss, placental abruption, preterm birth, premature rupture of membranes, cesarean delivery, preeclampsia and fetal growth restriction [4, 9, 11–13], while others failing to demonstrate any link [5, 10, 14, 15]. For example, a retrospective cohort study demonstrated that women with ultrasound-detected subchorionic hemorrhage before 22 weeks of gestation had an increased risk of placental abruption and preterm birth [11]. However, Yin et al. reported that the first-trimester SCH detected at 6–8 weeks of gestation was not associated with adverse pregnancy outcomes in singleton pregnancies after fresh embryo transfers [14]. The conflicting findings may be due to the differences in the sample size, the study design and the heterogeneous study population. In addition, some studies did not consider the impact of subchorionic hematoma accompanying symptoms such as abdominal pain and vaginal bleeding on outcomes, as well as few studies have investigated the effect of hematoma size and duration on pregnancy outcomes.

In light of the limitations of previous researches, the objective of this study was to investigate the association between the first-trimester asymptomatic subchorionic hematoma detected by ultrasound and the adverse pregnancy outcomes in singleton pregnancy of natural conception, and to further explore the effect of the duration and size of subchorionic hematoma on adverse pregnancy outcomes after adjustment for confounders.

## Methods

This was an observational retrospective cohort study including all the pregnant women who were diagnosed with subchorionic hematoma by ultrasound at their initial visits in the first trimester at our hospital from January 1, 2020 to December 31, 2020. Pregnant women without subchorionic hematoma who underwent ultrasonography during the same period were included as the control group. These women were matched with those in subchorionic hematoma group on baseline characteristics including age composition, body mass index (BMI), gravidity, parity, and gestational age (at first ultrasound).

All the enrolled participants were 20–45 years old with singleton pregnancy who concieved naturally. Exclusion criteria were women with assisted reproduction, reproductive tract infection, polycystic ovary, uterine malformation, COVID-19 infection, invasive placentation (placenta accreta spectrum), hysterectomy, with accompanying symptoms as vaginal bleeding or abdominal pain in early pregnancy and during follow-up, with history of chronic hypertension, intrahepatic cholestasis of pregnancy, hypertensive disorders of pregnancy, postpartum hemorrhage, adherent placenta, fetal growth restriction, and preterm birth, pregestational diabetes, prepregnancy hypothyroidism and hyperthyroidism, renal system disease, nutritional deficiency, immune system disease, hematological system diseases, and prepregnancy thrombocytopenia, or with incomplete clinical data, as well as neonates with stillbirth, multiples, or fetal malformations.

**Fig. 1:**
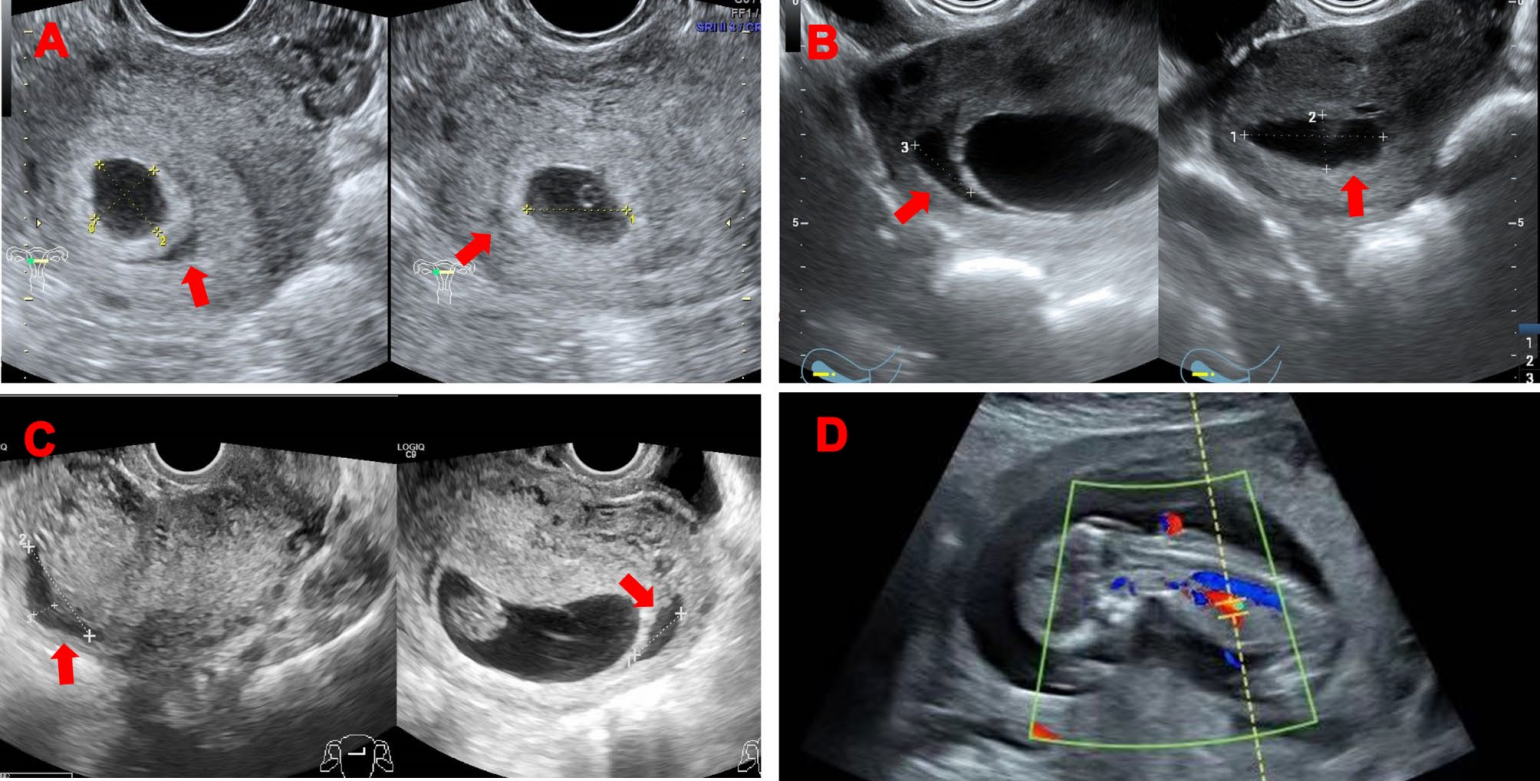
Ultrasound images of a single patient at different gestational ages in the subchorionic hematoma group. **A**: At 5 weeks and 4 days of gestation with subchorionic hematoma measuring 1.85*1.64*1.42 cm^3^. **B**: At 8 weeks and 1 days of gestation with subchorionic hematoma measuring 3.11*1.23*1.25 cm^3^. **C**: At 10 weeks and 1 days of gestation with subchorionic hematoma measuring 1.74*3.00*0.62 cm^3^. **D**: The subchorionic hematoma disappeared at 13 weeks and 1 days of gestation. *Arrows* indicate subchorionic hematoma. The hematoma size used for analysis in this study was the initial size of the hematoma measured at 5–10 weeks

Gestational age was determined by using the first day of the last normal regular menstrual period, and the first trimester ultrasound scan was used for dating in the pregnant women with irregular menstrual period. Subchorionic hematoma was defined as a crescent-shaped, echo-free area between the chorionic membrane and the myometrium [16]. For each patient, the initial ultrasound scan reports performed between 5 and 10 weeks of gestation were reviewed for the presence or the absence of a subchorionic hematoma and the size of subchorionic hematoma. Since most subchorionic hematoma showed crescentic and oblong shapes, the size of the hematoma was calculated by multiplying the three dimensions of the hematoma measured by ultrasound and dividing the product by two [17, 18]. The unit of subchorionic hematoma is cm^3^. The hematoma size used for analysis in this study was the initial size of the hematoma measured at 5–10 weeks. All patients with subchorionic hematoma were followed and reassessed accordingly, and they were assessed through ultrasound every 7–14 days until subchorionic hematoma disappeared (Fig. 1). They underwent three or four B-ultrasound examinations in the first trimester to monitor the changes of hematoma, as well as other indicators like detection of fetal nuchal translucency thickness. The duration of subchorionic hematoma was the time from the first detection of the hematoma by ultrasound to its disappearance. Women in the no subchorionic hematoma group underwent their first ultrasonography at 5–10 weeks of gestation. They also underwent two or three B-ultrasound examinations in the first trimester for their demand and detection of fetal nuchal translucency thickness.

We reviewed the computerized medical records to obtain the information of baseline maternal characteristics and related adverse pregnancy outcomes. These adverse complications and outcomes included spontaneous abortion (defined as the loss of pregnancy from natural causes before the 20th week of pregnancy with fetus weight < 500 g), gestational hypothyroidism (including overt hypothyroidism, defined as elevated serum thyroid-stimulating hormone (TSH) levels (> 2.5 mIU/L) in conjunction with decreased serum free thyroxine (FT4) or serum TSH levels > 10 mIU/L, irrespective of FT4 levels; and subclinical hypothyroidism, defined as elevated serum TSH levels between 2.5 and 10 mIU/L with normal serum FT4 levels; and isolated hypothyroxinemia, defined as a normal maternal TSH concentration in conjunction with FT4 concentrations in the lower 5th or 10th percentile of the reference range), intrahepatic cholestasis of pregnancy (ICP, defined as pruritus and an elevation in serum bile acid concentrations), premature rupture of membranes (PROM, defined as rupture of membranes prior to the onset of labor, which include term premature rupture of membranes (TPROM, defined as premature rupture of membranes happening after 37 weeks of gestation), and preterm premature rupture of membranes (PPROM, defined as premature rupture of membranes happening before 37 weeks of gestation)), gestational diabetes mellitus (GDM, defined as fasting plasma glucose (FPG) ≥ 5.1 mmol/L, or 1 h plasma glucose ≥ 10.0mmol/L, or 2 h plasma glucose ≥ 8.5 mmol/L at 24–28 weeks of gestation), oligohydramnios (defined as amniotic fluid volume ≤ 300ml, or the vertical depth of the maximum dark area of amniotic fluid under ultrasound was ≤ 2 cm or the amniotic fluid index was ≤ 5 cm in the third trimester of pregnancy), hypertensive disorders of pregnancy (HDP) (including gestational hypertension, defined as systolic blood pressure ≥ 140mmHg and/or diastolic blood pressure ≥ 90mmHg after 20 gestational weeks, and preeclampsia, defined as hypertension after 20 gestational weeks with proteinuria, uteroplacental dysfunction or organ damage, women with some conditions (the HELLP syndrome) related to HDP were also included in this group), gestational thrombocytopenia (defined as platelet count < 150 × 10^9^/L during the pregnancy), postpartum hemorrhage (defined as blood loss ≥ 500ml during vaginal birth, or ≥ 1000ml during cesarean section, within 24 h after delivery of the fetus), placenta adhesion (defined as pathologic adherence of the placenta diagnosed at time of delivery, which required manual placental extraction), intraamniotic infection (IAI) (defined by standard clinical criteria (maternal fever (≥ 38.0 °C) and ≥ 1 symptom (maternal tachycardia > 100 bpm, fetal tachycardia > 160 bpm, uterine tenderness, maternal leukocytosis > 15,000 cells/mm^3^, and/or foul odor of the amniotic fluid)) Moreover, placentae and gestational membranes were also reviewed for histologic evidence of infection by postpartum pathology), neonatal asphyxia (defined as low Apgar score or abnormal umbilical cord blood gas analysis or both), macrosomia (defined as birth weight ≥ 4000 g), fetal growth restricted (FGR, defined as birth weight less than the 10th percentile) and preterm birth (defined as gestational age ≥ 20 weeks and < 37 weeks (140–258 days)).

Data were analyzed using SPSS 26.0 software (SPSS, Chicago, IL, USA). Normally distributed variables were presented as mean ± standard deviation and were analyzed by Student’s t test. Nonnormally distributed variables were expressed as median and interquartile range and were analyzed by Mann-Whitney U test. Categorical variables were presented as number (%) and were analyzed by Pearson’s Chi-square test, univariate logistic regression and multivariate logistic regression. Multivariable logistic regression was used to control for differences in baseline characteristics between the two groups that were statistically significant in the univariable analysis (*P* < 0.05). Adjusted odds ratios with 95% CIs were estimated from the regression analysis. Sample sizes achieve 90% power to detect a difference of 0.9 between the null hypothesis with a significance level (alpha) of 0.05 using a two-sided two-sample test. Null hypothesis for this study was that there was no significant association between the presence, size or duration of a first-trimester asymptomatic subchorionic hematoma and pregnancy outcomes in women with singleton pregnancies. *P* value < 0.05 was considered to be statistically significant.

## Results

**Fig. 2:**
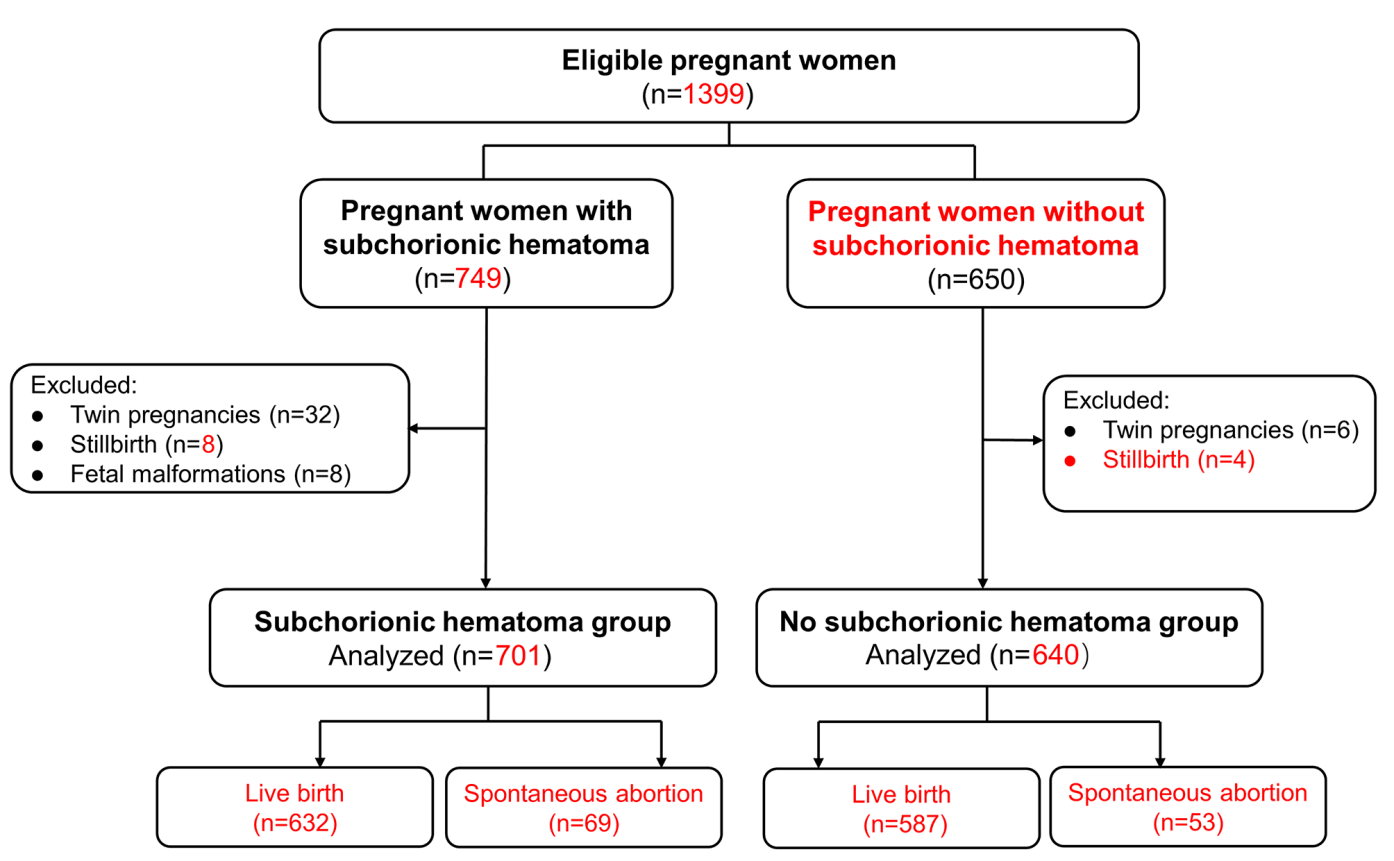
Flowchart

As shown in Fig. 2, a total of 1399 pregnant women met the inclusion criteria during the study period. We excluded 38 women with twin pregnancies, 12 women with stillbirth and 8 women with fetal malformations. Finally, 1341 women were eligible for the final analysis, 701 of whom had a subchorionic hematoma and 640 of whom did not have subchorionic hematoma. We first compared the spontaneous abortion rates between the two groups. Further adverse pregnancy outcomes were compared among the remaining population with live births, including 632 cases in subchorionic hematoma group and 587 cases in no subchorionic hematoma group. Baseline characteristics of the subchorionic hematoma group and no subchorionic hematoma group were provided in Table 1. There were no differences in baseline characteristics between the two groups including maternal age, pre-pregnancy BMI, gravidity, parity, rate of previous abortion, gestational age at first ultrasound and number of previous abortions (*P* > 0.05).

**Table 1:** Baseline Maternal Characteristics Based on the Presence or Absence of a Subchorionic Hematoma

Characteristic	Subchorionic Hematoma (n = 632)	No Subchorionic Hematoma (n = 587)	*P*
Maternal age (year)	28.76 ± 4.04	28.88 ± 4.27	0.620
Pre-pregnancy BMI (kg/m2)	20.70 (19.00, 22.90)	20.50 (19.00, 22.10)	0.169
Gravidity	2 (1, 3)	2 (1, 3)	0.137
Parity	1 (1, 2)	1(1, 2)	0.659
Number of previous abortions	0 (0, 1)	0 (0, 1)	0.085
Gestational age at first B-ultrasound (week)	6.72 (6.57, 7.86)	6.86 (6.14, 7.43)	0.083

Data were presented as mean ± SD or median (IQR).

abortions: referred to pregnancy loss before 20 weeks and included spontaneous abortion, elective abortion and ectopic pregnancies

### Perinatal outcomes in subchorionic hematoma pregnancies versus no subchorionic hematoma pregnancies

As shown in Table 2, in comparison with no subchorionic hematoma population, women with subchorionic hematoma in the first trimester had a significantly increased risk of GDM (*P* = 0.045), gestational thrombocytopenia ( *P* < 0.001), postpartum hemorrhage (*P* = 0.050) and placenta adhesion (*P* < 0.001), but no increased risk of spontaneous abortion, gestational hypothyroidism, ICP, TPROM, PPROM, HDP, oligohydramnios and IAI (*P* > 0.05). Newborns born to mothers diagnosed with a subchorionic hematoma in early pregnancy were more likely to develop FGR (*P* = 0.001) and macrosomia (*P* < 0.001) than those born to normal pregnant mothers without a subchorionic hematoma. There were no differences in the incidence of neonatal asphyxia, 1-min Apgar score ≤ 7 and preterm birth between the two groups (*P* > 0.05).

**Table 2:** Effect of the presence of subchorionic hematoma in first trimester on pregnancy outcomes

	Subchorionic Hematoma (n = 701)	No Subchorionic Hematoma (n = 640)	OR (95%CI)	*P*
Spontaneous abortion	69 (9.8)	53 (8.3)	1.209 (0.831–1.760)	0.321
**Mothers with live birth**	(n = 632)	(n = 587)		
Gestational hypothyroidism	69 (10.9)	67 (11.4)	0.951 (0.666–1.359)	0.783
ICP	16 (2.5)	11 (1.9)	1.360 (0.626–2.955)	0.436
TPROM	115 (18.2)	87 (14.8)	1.278 (0.943–1.733)	0.113
PPROM	15 (2.4)	8(1.4)	1.760 (0.740–4.181)	0.195
GDM	133 (21.0)	114 (19.4)	1.106 (1.006–1.761)	0.045^a^
HDP	48 (7.6)	31 (5.3)	1.474 (0.925–2.350)	0.101
Oligohydramnios	47 (7.4)	31 (5.3)	1.441 (0.902–2.301)	0.124
Gestational thrombocytopenia	32 (5.1)	8 (1.4)	3.860 (1.764–8.447)	< 0.001^a^
Postpartum hemorrhage	28 (4.4)	14 (2.4)	1.897 (0.989–3.640)	0.050
Placenta adhesion	57 (9.0)	21 (3.6)	2.672 (1.599–4.465)	＜0.001^a^
IAI	232 (36.8)	204 (34.8)	1.092 (0.863–1.380)	0.464
**Neonates**	(n = 632)	(n = 587)		
Neonatal asphyxia	4 (0.6)	3 (0.5)	1.240 (0.276–5.563)	> 0.999
1-min Apgar score (≤ 7)	4 (0.6)	3 (0.5)	1.240 (0.276–5.563)	> 0.999
FGR	26 (4.1)	6 (1.0)	4.155 (1.698–10.167)	0.001^a^
Macrosomia	34 (5.4)	4 (0.7)	8.287 (2.922–23.499)	＜0.001^a^
Preterm birth	34 (5.4)	32 (5.5)	0.986 (0.600–1.620)	0.956

ICP: Intrahepatic cholestasis of pregnancy; TPROM: Term premature rupture of membranes; PPROM: Preterm premature rupture of membranes; GDM: Gestational diabetes mellitus; HDP: Hypertensive disorders of pregnancy; IAI: Intraamniotic infection; FGR: Fetal growth restriction

OR: crude odds ratio

Data were presented as n (%)

^a^: *P*＜0.05

### Relationship between the size of subchorionic hematoma and perinatal outcomes

To investigate the impact of hematoma size on pregnancy outcomes in pregnant women with subchorionic hematoma, the size of the hematoma determined through the initial ultrasound was utilized as the independent variable, whereas the pregnancy outcome served as the dependent variable. In addition, to address potential confounding factors, a multivariate regression analysis was conducted, encompassing these variables within the analysis. As shown in Table 3, after adjusting for maternal age, pre-pregnancy BMI, gravidity, parity, number of previous abortions, gestational age at first ultrasound and gestational age at delivery, the larger size of subchorionic hematoma was associated with a higher risk of gestational hypothyroidism (adjusted odds ratio (aOR) [95%CI]: 1.029[1.004–1.054], *P* = 0.021), ICP (aOR [95%CI]: 1.095[1.047–1.146], *P* < 0.001), TPROM (aOR [95%CI]: 1.044(1.005–1.085), *P* = 0.029), HDP (aOR [95%CI]: 1.030[1.0004-1.060], *P* = 0.048), gestational thrombocytopenia (aOR [95%CI]: 1.078[1.045–1.113], *P* < 0.001), placenta adhesion (aOR [95%CI]: 1.054 [1.027–1.082], *P* < 0.001) in pregnant women, Furthermore, there’s also a significant association between the size of subchorionic hematoma in early pregnancy and the prevalence of FGR in neonates (aOR [95%CI]: 1.028[1.009–1.063], *P* < 0.001). But there was no association between the size of subchorionic hematoma and the incidence of spontaneous abortion, PPROM, GDM, oligohydramnios, postpartum hemorrhage, IAI, neonatal asphyxia, 1-min Apgar score ≤ 7, macrosomia and preterm birth (*P* > 0.05).

**Table 3:** Effect of the size of subchorionic hematoma in first trimester on pregnancy outcomes

	N (%) (n = 701)	OR (95%CI)	*P*	Adjusted OR (95%CI)	*P*
Spontaneous abortion	69 (9.8)	0.964 (0.916–1.013)	0.149	0.975 (0.927–1.025)	0.314
**Mothers with live birth**	(n = 632)				
Gestational hypothyroidism	69 (10.9)	1.048 (1.006–1.091)	0.025	1.029 (1.004–1.054)	0.021^a^
ICP	16 (2.5)	1.067 (1.013–1.123)	0.013	1.095 (1.047–1.146)	＜0.001^a^
TPROM	115 (16.3)	1.036(0.998–1.075)	0.066	1.044(1.005–1.085)	0.029^a^
PPROM	15 (2.1)	0.956(0.802–1.139)	0.614	0.972(0.809–1.169)	0.766
GDM	133 (21.0)	0.960 (0.902–1.022)	0.205	0.986 (0.956–1.018)	0.383
HDP	48 (7.6)	1.011 (0.955–1.070)	0.714	1.030 (1.0004-1.060)	0.048^a^
Oligohydramnios	47 (7.4)	0.999 (0.935–1.067)	0.970	1.000 (0.967–1.034)	0.994
Gestational thrombocytopenia	32 (5.1)	1.072 (1.023–1.123)	0.004	1.078 (1.045–1.113)	＜0.001^a^
Postpartum hemorrhage	28 (4.4)	1.010 (0.939–1.086)	0.791	1.004(0.966–1.094)	0.842
Placenta adhesion	57 (9.0)	1.084 (1.037–1.134)	0.000	1.054 (1.027–1.082)	＜0.001^a^
IAI	232 (36.7)	1.013 (0.979–1.049)	0.454	1.007 (0.989–1.024)	0.470
**Neonates**	(n = 632)				
Neonatal asphyxia	4 (0.6)	1.020 (0.871–1.194)	0.807	1.021 (0.929–1.122)	0.668
1-min Apgar score (≤ 7)	4 (0.6)	1.020 (0.871–1.194)	0.807	1.021 (0.929–1.122)	0.668
FGR	26 (4.1)	1.066 (1.017–1.118)	0.008	1.028 (1.009–1.063)	＜0.001^a^
Macrosomia	34 (5.4)	0.999 (0.964–1.036)	0.960	1.000 (0.962–1.039)	0.991
Preterm birth	34 (5.4)	1.001 (0.929–1.078)	0.977	0.999 (0.961–1.038)	0.962

OR: crude odds ratio; ICP: Intrahepatic cholestasis of pregnancy; TPROM: Term premature rupture of membranes; PPROM: Preterm premature rupture of membranes; GDM: Gestational diabetes mellitus; HDP: Hypertensive disorders of pregnancy; IAI: Intraamniotic infection; FGR: Fetal growth restriction

Data were presented as n (%)

aOR: Adjusted odds ratio for maternal age (year), pre-pregnancy BMI (kg/m^2^), gravidity, parity, gestational age at first B-ultrasound (week), number of previous abortions, gestational age at delivery (week)

^a^: *P*＜0.05

Furthermore, the adverse pregnant outcomes associated with the initial hematoma size in Table 3 were analyzed to explore possible thresholds of the hematoma size for these adverse pregnancy outcomes. As shown in Table 4, we employed a stratification approach based on quartiles of hematoma size for the group of pregnant women with hematoma. Then separate comparisons were conducted between each quartile group and pregnant women without hematoma. Additionally, to address potential confounding factors, we performed a multivariable regression analysis to account for their influence on the outcomes. Among subchorionic hematoma detected in this study, the median (interquartile range) of hematoma size at initial diagnosis was 0.9900 (0.4190, 2.4390) cm3. According to the median (interquartile range) size, the pregnant women with SCH were divided into less than 25%ile group, 25%ile-50%ile group, 50%ile-75%ile group and more than 75%ile group. As shown in Table 4, when the initial subchorionic hematoma size was greater than larger than 0.9900 cm3 and smaller than 2.4390 cm3 (50-75%), for each initial hematoma size in cm^3^, the odds of gestational thrombocytopenia was 3.386-fold higher (aOR [95%CI]: 3.386[1.142–10.041], *P* = 0.028), the odds of placenta adhesion was 2.343-fold higher (aOR [95%CI]: 2.343[1.087–5.053], *P* = 0.030), the odds of FGR was 5.314-fold higher (aOR [95%CI]: 5.314[1.705–16.560], *P* = 0.004). When the initial subchorionic hematoma size was greater than the 75%ile (2.4390 cm3) at the initial 5–10 weeks ultrasound, for each unit increase in cm^3^-volume of the initial subchorionic hematoma, the adjusted odds of ICP increase by a factor of 3.085 (aOR [95%CI]: 3.085[1.188–8.010], *P* = 0.021), the adjusted odds of TPROM increased by a factor of 1.581 (aOR [95%CI]: 1.581[1.004–2.487], *P* = 0.048), the adjusted odds of HDP increased by a factor of 1.864 (aOR [95%CI]: 1.864[0.950–3.656], *P* = 0.070), the adjusted odds of gestational thrombocytopenia increased by a factor of 10.685 (aOR [95%CI]: 10.685[4.483–25.469], *P* < 0.001), the adjusted odds of placenta adhesion increased by a factor of 6.817 (aOR [95%CI]: 6.817[3.667–12.673], *P* < 0.001), the adjusted odds of FGR increased by a factor of 10.211 (aOR [95%CI]: 10.211[3.643–28.623], *P* < 0.001). Therefore, in comparison with no subchorionic hematoma women, the risks of ICP, TPROM, HDP, gestational thrombocytopenia, placenta adhesion and FGR were significantly increased in pregnant women with the size of SCH larger than 2.4390 cm^3^ in early pregnancy.

**Table 4:** Effect of the size of subchorionic hematoma in first trimester on pregnancy outcomes

	No hematoma (n = 587)	＜25%ile (< 0.4190cm^3^) (n = 152)	25-50%ile (0.4190-0.9900cm^3^) (n = 159)	50-75%ile (0.9900-2.4390cm^3^) (n = 155)	＞75%ile (> 2.4390cm^3^) (n = 166)
		aOR (95%CI) *P*	aOR (95%CI) *P*	aOR (95%CI) *P*	aOR (95%CI) *P*
**Mothers with live birth**					
Gestational hypothyroidism	1(Reference)	0.857(0.474–1.550) 0.610	0.829(0.459–1.498) 0.535	1.203(0.702–2.061) 0.502	1.032(0.597–1.784) 0.911
ICP	1(Reference)	0.631(0.133–3.005) 0.563	0.352(0.045–2.779) 0.322	1.411(0.426–4.673) 0.573	3.085(1.188–8.010) 0.021^a^
TPROM	1(Reference)	1.536(0.965–2.446) 0.070	0.692(0.399-1.200) 0.190	1.206(0.747–1.945) 0.444	1.581(1.004–2.487) 0.048^a^
HDP	1(Reference)	1.287(0.610–2.713) 0.508	1.866(0.957–3.639) 0.067	1.301(0.616–2.746) 0.491	1.864(0.950–3.656) 0.070^a^
Gestational thrombocytopenia	1(Reference)	1.983(0.585–6.721) 0.272	1.413(0.365–5.474) 0.617	3.386(1.142–10.041) 0.028 ^a^	10.685(4.483–25.469) ＜0.001^a^
Placenta adhesion	1(Reference)	1.421(0.608–3.320) 0.418	1.715(0.761–3.863) 0.193	2.343(1.087–5.053) 0.030 ^a^	6.817(3.667–12.673) ＜0.001^a^
**Neonates**					
FGR	1(Reference)	1.847(0.441–7.735) 0.401	1.994(0.487–8.167) 0.337	5.314(1.705–16.560) 0.004 ^a^	10.211(3.643–28.623) ＜0.001^a^

ICP: Intrahepatic cholestasis of pregnancy; TPROM: Term premature rupture of membranes; HDP: Hypertensive disorders of pregnancy; FGR: Fetal growth restriction

aOR: Adjusted odds ratio for maternal age (year), pre-pregnancy BMI (kg/m^2^), gravidity, parity, gestational age at first B-ultrasound (week), number of previous abortions, gestational age at delivery (week)

^a^: *P*＜0.05

### Relationship between duration of subchorionic hematoma and perinatal outcomes

To investigate the impact of the duration of hematoma on pregnancy outcomes in pregnant women with subchorionic hematoma, the duration of the hematoma determined through the continuous ultrasound was utilized as the independent variable, whereas the pregnancy outcome served as the dependent variable. In addition, to address potential confounding factors, a multivariate regression analysis was conducted, encompassing these variables within the analysis. Table 5 described the association between the duration of subchorionic hematoma and the perinatal outcomes. Multivariate logistic regression showed that the longer duration of the hematoma was associated with higher odds of TPROM (aOR [95%CI]: 1.070[1.027–1.115], *P* = 0.001), GDM (aOR [95%CI]: 1.938 [1.886–1.993]), *P* = 0.027) and FGR (aOR [95%CI]: 1.194 [1.124–1.268], *P* < 0.001), after adjusting for maternal age, pre-pregnancy BMI, gravidity, parity, number of previous abortions, gestational age at first ultrasound and gestational age at delivery. However, the duration of subchorionic hematoma had no association with risks of gestational hypothyroidism, ICP, PPROM, HDP, oligohydramnios, gestational thrombocytopenia, postpartum hemorrhage, placenta adhesion, IAI, neonatal asphyxia, 1-min Apgar score ≤ 7, macrosomia and preterm birth (*P* > 0.05).

**Table 5:** Effect of the duration of subchorionic hematoma in first trimester on pregnancy outcomes

	N (%) (n = 632)	OR (95%CI)	*P*	Adjusted OR (95%CI)	*P*
**Mothers with live birth**					
Gestational hypothyroidism	69 (10.9)	1.017 (0.965–1.071)	0.530	1.024 (0.972–1.079)	0.370
ICP	16 (2.5)	1.008 (0.907–1.121)	0.882	1.018 (0.915–1.133)	0.745
TPROM	115 (16.3)	1.065(1.023–1.108)	0.002 ^a^	1.070(1.027–1.115)	0.001^a^
PPROM	15 (2.1)	0.901(0.766–1.059)	0.204	0.927(0.794–1.082)	0.927
GDM	133 (21.0)	1.940 (1.890–1.992)	0.025 ^a^	1.938 (1.886–1.993)	0.027^a^
HDP	48 (7.6)	0.991 (0.927–1.060)	0.790	0.999 (0.933–1.071)	0.986
Oligohydramnios	47 (7.4)	1.048 (0.992–1.108)	0.095	1.054 (0.997–1.114)	0.065
Gestational thrombocytopenia	32 (5.1)	0.996 (0.919–1.078)	0.914	1.009 (0.931–1.093)	0.831
Postpartum hemorrhage	28 (4.4)	0.914 (0.815–1.026)	0.126	0.916 (0.815–1.028)	0.137
Placenta adhesion	57 (9.0)	1.029 (0.975–1.086)	0.301	1.036 (0.980–1.096)	0.207
IAI	232 (36.7)	0.964 (0.928–1.001)	0.060	0.968 (0.931–1.006)	0.099
**Neonates**					
Neonatal asphyxia	4 (0.6)	0.975 (0.767–1.241)	0.839	0.971 (0.738–1.278)	0.833
1-min Apgar score (≤ 7)	4 (0.6)	0.975 (0.767–1.241)	0.839	0.971 (0.738–1.278)	0.833
FGR	26 (4.1)	1.195 (1.127–1.267)	＜0.001 ^a^	1.194 (1.124–1.268)	＜0.001^a^
Macrosomia	34 (5.4)	1.036 (0.970–1.107)	0.289	1.042 (0.970–1.120)	0.255
Preterm birth	34 (5.4)	0.991 (0.916–1.073)	0.828	0.998 (0.923–1.080)	0.967

OR: crude odds ratio; ICP: Intrahepatic cholestasis of pregnancy; TPROM: Term premature rupture of membranes; PPROM: Preterm premature rupture of membranes; GDM: Gestational diabetes mellitus; HDP: Hypertensive disorders of pregnancy; IAI: Intraamniotic infection; FGR: Fetal growth restriction

Data were presented as n (%)

aOR: Adjusted odds ratio for maternal age (year), pre-pregnancy BMI (kg/m^2^), gravidity, parity, gestational age at first B-ultrasound (week), number of previous abortions, gestational age at delivery (week)

^a^: *P*＜0.05

Furthermore, the adverse pregnant outcomes associated with the duration of hematoma in Table 5 were analyzed to explore the possible thresholds of the duration of hematoma for these adverse pregnancy outcomes. As shown in Table 6, we employed a stratification approach based on quartiles of the duration of hematoma for the group of pregnant women with hematoma. Then separate comparisons were conducted between each quartile group and pregnant women without hematoma. Additionally, to address potential confounding factors, we performed a multivariable regression analysis to account for their influence on the outcomes. The median (interquartile range) duration of SCH was 5.43 (2.86, 6.86) weeks. According to the median (interquartile range) duration, the pregnant women with SCH were divided into less than 25%ile group, 25%ile-50%ile group, 50%ile − 75%ile group and more than 75%ile group. As shown in Table 6, when the duration of subchorionic hematoma was longer than the 75%ile (6.86 weeks), the adjusted odds of TPROM increased by a factor of 1.874 for each unit increased in week-duration of subchorionic hematoma (aOR [95%CI]: 1.874[1.225–2.867] *P* = 0.004), the adjusted odds of GDM increased by a factor of 2.256 for each unit increase in week-duration of subchorionic hematoma (aOR [95%CI]: 2.256[1.343–3.787], *P* = 0.002), the adjusted odds of FGR increased by a factor of 13.355 for each unit increase in week-duration of subchorionic hematoma (aOR [95%CI]: 13.355[5.114–34.874], *P*＜0.001). Therefore, in comparison with no subchorionic hematoma women, the risks of TPROM, GDM and FGR were significantly increased in pregnant women with the duration of SCH longer than 6.86 weeks.

**Table 6:** Effect of the duration of subchorionic hematoma in first trimester on pregnancy outcomes

	No hematoma (n = 587)	＜25%ile(< 2.86w) (n = 152)	25-50%ile (2.86-5.43w) (n = 159)	50-75%ile (5.43-6.86w) (n = 155)	＞75%ile (> 6.86w) (n = 166)
		aOR (95%CI) *P*	aOR (95%CI) *P*	aOR (95%CI) *P*	aOR (95%CI) *P*
**Mothers with live birth**					
TPROM	1(Reference)	0.934(0.523–1.668) 0.818	0.881(0.532–1.460) 0.623	1.152(0.712–1.864) 0.564	1.874(1.225–2.867) 0.004^a^
GDM	1(Reference)	0.920(0.553–1.531) 0.749	0.800(0.500-1.279) 0.350	1.099(0.724–1.669) 0.657	2.256(1.343–3.787) 0.002^a^
**Neonates**					
FGR	1(Reference)	0.925(0.109–7.842) 0.943	1.115(0.216–5.748) 0.895	1.895(0.458–7.840) 0.378	13.355(5.114–34.874) ＜0.001^a^

TPROM: Term premature rupture of membranes; GDM: Gestational diabetes mellitus; FGR: Fetal growth restriction

aOR: Adjusted odds ratio for maternal age (year), pre-pregnancy BMI (kg/m^2^), gravidity, parity, gestational age at first B-ultrasound (week), number of previous abortions, gestational age at delivery (week)

^a^: *P*＜0.05

## Discussion

### Main findings

In the present study, we found that the presence of a first-trimester asymptomatic subchorionic hematoma in singleton pregnancies was associated with adverse pregnancy outcomes including GDM, gestational thrombocytopenia, placenta adhesion, FGR, macrosomia, preterm birth at later gestations. Most importantly, the size of hematoma was positively associated with the occurrence of gestational hypothyroidism, ICP, TPROM, gestational thrombocytopenia, placenta adhesion and FGR, and the duration of hematoma was positively associated with the incidence of FGR, TPROM, and GDM. Furthermore, the stratified analysis according to the size and the duration of hematoma indicated that a hematoma larger than 2.4390 cm^3^ detected by B-ultrasound at 5–10 weeks of gestation persisted for about 7 weeks, which was strongly associated with TPROM and FGR.

Our study confirmed the findings of some studies [4, 10, 13, 19] that the presence of a hematoma was associated with an increased risk for fetal growth restriction, PROM and placenta adhesion. Many of these pregnancy complications fall under the rubric of placental dysfunction [7, 8, 20]. Poor placentation can damage angiogenesis which results in the formation of weak vessels that easily tear [5, 6]. Although the exact mechanism remains unclear, subchorionic hematoma is believed to the result from ruptured blood vessels during the process of abnormal placental implantation [4, 11, 12]. Thus, the subchorionic hematoma associated with the perinatal complications may be due to poor placenta, and it is a possible early ultrasound marker of abnormal placentation.However, inconsistent with the findings of previous studies [4, 13], our data did not show an association between the subchorionic hematoma and HDP, another condition associated with poor placental function. This may be due to the selection bias caused by our study’s exclusion of women at high risk for hypertensive pregnancies with a history of hypertension or immune system disease. In addition, pregnant women with subchorionic hematoma in the first trimester had changes in vaginal flora in the second trimester [21], which may be the reasons for the predisposition of some complications such as TPROM and placenta adhesion.

Additionally, our results showed that newborns born to mothers diagnosed with a subchorionic hematoma in early pregnancy were more likely to develop FGR and macrosomia than those born to normal pregnant mothers without a subchorionic hematoma. We propose a hypothesis as to why the presence of subchorionic hematoma appeared to have an association with both FGR and macrosomia. Mothers with small subchorionic hematoma size may have macrosomia because they have reduced movement to prevent miscarriage, while mothers with large subchorionic hematoma size may have fetal growth restriction due to the poor placental function. Our study focused on the associations between the size and the duration of subchorionic hematoma and pregnancy outcomes in pregnant women, with the result that the size and the duration of a first-trimester asymptomatic subchorionic hematoma may be associated with fetal growth restriction but not macrosomia.

It has been proposed that subchorionic hematoma after vaginal bleeding consumes coagulation factor XIII, and the presence of a subchorionic hematoma may be the first indicator of an underlying thrombophilia [22, 23], which may explain why the pregnant women with subchorionic hematoma are prone to gestational thrombocytopenia and postpartum hemorrhage. Moreover, placenta adhesion associated with subchorionic hematoma is also one of the important causes of postpartum hemorrhage.

Premature perfusion of the intervillous space as occurs with subchorionic hemorrhage may be a possible mechanism leading to pregnancy loss [5, 24]., which points to a correlation between the subchorionic hematoma and pregnancy loss. In addition, mechanical or inflammatory uterine irritation due to localized accumulation of blood can stimulate contractions, which may lead to preterm birth [1, 25]. Preterm birth is the consequence of four main mechanisms: activation of the maternal-fetal placental interaction with the hypothalamic-pituitary-adrenal axis, inflammation in the amniochorionic-decidual tissue, decidual hemorrhage and pathological distention of the myometrium [26]. So there are many causes of preterm birth including infection, uterine malformation, polyhydramnios, twins, fetal malformations, mothers with nutritional deficiency, immune system disease, diabetes or hypertension. To reduce the confounders for the observed results, our study excluded women with these contributing factors to preterm birth. Thus, the rate of preterm birth was low whether in the subchorionic hematoma group or in the non-subchorionic hematoma group, and there was no difference between the two groups. We did not find the link between the subchorionic hematoma and preterm birth.

However, the results of recent studies as to the relationship between subchorionic hematoma and pregnancy loss or preterm birth were inconsistent. Several studies found an increased rate of spontaneous abortion and preterm birth among women with subchorionic hematoma [3, 5, 6, 9, 11, 12, 19], while others and ours did not find the link [1, 10, 14, 22, 27]. Many factors contributed to the contradictory results including study designs, study subjects, lack of important clinical information, and inability to control for important covariates that may influence the rate of adverse pregnancy outcomes. One of the most important reasons for this discrepancy was the different study populations involved. Some researches focused on infertile women with embryo transfers or other assisted reproductive techniques who may be accompanied by the vaginal bleeding [14, 15], while our study focused on the asymptomatic subchorionic hematoma women without assisted reproduction.

Doppler studies have shown a significant relationship between enlarged hematoma and reduced blood flow velocities in spiral arteries, potentially threatening the continuation of pregnancy through a direct pressure-volume effect [9, 28]. Adverse pregnancy outcomes were more likely to occur in women with a large hematoma than those with a medium-or small-sized hematoma (the sonographic evaluation involved examining the size of the hematoma relative to the gestational sac size, classifying them as small (less than 20%), medium (20–50%), or large (more than 50%)) [19, 29, 30], which was supported by our results. But no significant correlation of the hematoma size and adverse pregnancy outcomes was found in some studies [13, 27]. This difference may be due to the fact that we have given an “absolute” value for the hematoma, without relating it to the size of the gestational sac. Perhaps the presence and location of the hematoma that represents damage to the placenta, rather than its size, is important to pregnancy outcomes. It is difficult to draw conclusions from these conflicting results.

Another explanation for these discrepancies from available studies on hematoma volume and pregnancy outcome was the physiopathological mechanism of formation of the subchorionic hematoma. The size of the hematoma was determined by the amount of subchorionic bleeding, reabsorbed, and lost through the cervix. In particular, the amount of the bleeding through the cervix was difficult to calculate. Therefore, the size of the hematoma in those patients with vaginal bleeding may not represent a reliable estimation of the severity of the overall process. Our study focused on pregnant women with asymptomatic subchorionic hemorrhage detected by ultrasound, and revealed that subchorionic hematoma size was associated with FGR and TPROM at later gestations. This is important information for counseling patients, as the diagnosis of a subchorionic hematoma can cause significant concern among expectant parents.

Moreover, the relationships of the gestational hypothyroidism and ICP with the size of subchorionic hematoma were shown in our study. To our knowledge, no studies have reported this correlation, and the specific mechanism is unclear. The size of the hematoma does seem to have a clinical significance. Those pregnancies with large subchorionic hematoma warrant greater surveillance. In addition, large prospective randomized studies are required to explore the true role of subchorionic hematoma size in the prognosis of ongoing pregnancies.

Little research has been done on the relationship between the duration of subchorionic hematoma and adverse pregnancy outcomes. Pregnant women who develop subchorionic hematoma after vaginal bleeding have a longer duration of hematoma and are more likely to have adverse perinatal outcomes such as oligohydramnios and fetal distress [23]. It was reported that hematoma disappeared by the end of the first trimester [31] or 90.7% of subchorionic hematoma resolved by the second trimester [27]. Mackenzie et al. found that subchorionic hematoma persistence into the second trimester was not associated with an increased risk of adverse pregnancy outcomes [27]. However, our study found that the duration of asymptomatic subchorionic hematoma was positively associated with some adverse perinatal outcomes such as FGR, TPROM, and GDM, although most hematoma disappeared during the first trimester. Therefore, the persistence of the subchorionic hematoma may also be a predictor of adverse pregnancy outcomes. Prospective, multicenter, large-scale trials are needed to provide more evidence.

### Strengths and limitations

The strength of our study was that our data focused on pregnant women with asymptomatic subchorionic hematoma in early pregnancy, which could rule out the effect of some symptoms such as vaginal bleeding and abdominal pain on adverse pregnancy outcomes. Furthermore, we assessed the relationship between the size and duration of hematoma and a range of adverse outcomes during pregnancy and found that both were associated with FGR and TPROM. Additionally, we were able to control for confounding factors that might influence the outcomes such as maternal age, BMI, gravidity, parity, history of abortion and gestational age (at first ultrasound or at delivery). Our study was limited by its retrospective design and the data from one obstetric practice, rather than a more heterogeneous population. Another limitation of this study was that the shape of the subchorionic hematoma was irregular, thus, calculating the size of a hematoma may not be accurate enough. Finally, we did not consider the effect of hematoma location on pregnancy outcomes.

## Conclusions

In conclusion, the presence, size and duration of asymptomatic subchorionic hematoma detected at 5–10 weeks gestational age were associated with several adverse obstetric outcomes such as term premature rupture of membranes and fetal growth restriction.

## Data Availability

The datasets used and/or analysed during the current study are available from the corresponding author on reasonable request.

## Abbreviations

BMI: Body mass index
FGR: Fetal growth restricted
FPG: Fasting plasma glucose
FT4: Free thyroxine
GDM: Gestational diabetes mellitus
HDP: Hypertensive disorders of pregnancy
IAI: Intraamniotic infection
ICP: Intrahepatic cholestasis of pregnancy
IUGR: Intrauterine growth restriction
OR: Odds ratios
PROM: Premature rupture of membranes
TPROM: Term premature rupture of membranes
TSH: Thyroid-stimulating hormone
PPROM: Preterm premature rupture of membranes
SCH: Subchorionic hematoma
